# Treatment for non-small-cell lung cancer and circulating tumor cells

**DOI:** 10.2217/lmt-2017-0019

**Published:** 2018-06-22

**Authors:** Joel Mason, Benjamin Blyth, Michael P MacManus, Olga A Martin

**Affiliations:** 1 Division of Radiation Oncology, Peter MacCallum Cancer Centre, Melbourne, Australia; 2 Research Division, Peter MacCallum Cancer Center, Melbourne, Australia; 3 Department of Pathology, The University of Melbourne, Melbourne, Australia; 4 Peter MacCallum Department of Oncology, The University of Melbourne, Melbourne, Australia

## Abstract

Surgery is the main curative therapy for patients with localized non-small-cell lung cancer while radiotherapy (RT), alone or with concurrent platinum-based chemotherapy, remains the primary curative modality for locoregionally advanced non-small-cell lung cancer. The risk of distant metastasis is high after curative-intent treatment, largely attributable to the presence of undetected micrometastases, but which could also be related to treatment-related increases in circulating tumor cells (CTCs). CTC mobilization by RT or systemic therapies might either reflect efficient tumor destruction with improved prognosis, or might promote metastasis and thus represent a potential therapeutic target. RT may induce prometastatic biological alterations in CTC at the cellular level, which are detectable by ‘liquid biopsies’, though their rarity represents a major challenge. Improved methods of isolation and *ex vivo* propagation will be essential for the future of CTC research.

**Practice points**

- Lung cancer is the most common form of cancer diagnosed worldwide, still with a relatively poor survival rate.
- Lung cancer has a high rate of occurrence of post-treatment distant metastasis.
- Tumor cell entry into the vasculature part of the metastatic process may sometimes be influenced by therapeutic interventions.
- The presence and properties of circulating tumor cells (CTCs) during and after cancer treatment may have prognostic implications, not only for tumor response, but also for the development of metastasis and overall survival.
- We review current methodologies of CTC isolation and *ex vivo* propagation.
- Observational clinical trials are required to serially monitor CTC numbers and characteristics during and after cancer treatment and to correlate these changes with imaging and clinical findings to better understand their significance.

## Curative-intent treatment for non-small-cell lung cancer

Lung cancer is the most commonly diagnosed cancer worldwide, with one in 18 men and one in 51 women diagnosed with lung cancer before the age of 80 [1]. Each year over 1.6 million people are diagnosed with lung cancer and due to the poor survival rates a similar number die from the disease [2,3], the majority of these in the developing world.

Surgery is the standard treatment for resectable stage I and II non-small-cell lung cancer (NSCLC) with 5-year survival rates of 60–80% and 30–50% reported for stage I and II NSCLC, respectively [4]. Adjuvant chemotherapy can modestly improve outcomes in stage II disease. Medically unresectable stage I disease can be very effectively treated with stereotactic ablative body radiation therapy (SABR). For most patients with stage IIIA and IIIB disease, with disease encompassable within a tolerable radiation treatment volume, the most effective management is concurrent chemoradiation with curative intent. Management of stage IIIA patients with resectable N2 disease remains controversial. A combination of chemotherapy and radiotherapy (ChemoRT), or surgical resection (lobectomy and nodal dissection) after neoadjuvant chemotherapy or ChemoRT can all be effective.

The widely used radiotherapy (RT) fractionation schedule for locoregionally advanced NSCLC of 60 Gy in 30 fractions in 6 weeks became established after a trial comparing different doses was reported by Perez *et al*. [5]. This dose achieved lower local failure rates than continuous RT fractionation to 40 or 50 Gy, or 40 Gy delivered in split-course fractionation. A conventionally fractionated dose of approximately 60 Gy, combined with platinum-based chemotherapy, is effectively a standard of care, especially since the RTOG 0617 trial showed worse outcomes in patients treated to the higher dose of 74 Gy [6,7]. Hyperfractionation, whereby a larger number of lower-dose fractions are applied to a similar total dose of conventional RT, has also been employed. In a randomized clinical trial [8] treatment with 1.2 Gy fractions delivered twice daily to a total of 69.6 Gy was compared with conventional RT and conventional chemoradiation. ChemoRT gave the greatest 1-year survival rates (60%) when compared with conventional and hyperfractionated RT (46 and 51%, respectively, although long-term survival was similar). The results of ChemoRT have improved over the years with the adoption of concurrent platinum-based chemotherapy and the widespread adoption of ^18^F-fluorodeoxyglucose PET (FDG-PET) scanning for staging, patient selection and RT treatment planning. A further exciting development is the enormous improvement in progression-free survival that was recently reported with immunotherapy with darvalumab in patients who obtained a response to ChemoRT for NSCLC [9].

SABR is becoming increasingly used for patients with unresectable stage I primary tumors and oliogometastasis. This modality employs extremely high doses of radiation delivered to the primary tumor with steep-dose gradients from normal tissues, thereby maximizing tumor control and minimizing normal tissue toxicity [10]. A frequently used treatment schedule is 54 Gy in three fractions of 18 Gy. Many retrospective studies and preliminary results of randomized trials indicate that for unresectable stage I NSCLC patients, the application of SABR achieves better rates of overall survival and local control [11–13].

The overall poor 5-year survival rate of patients with NSCLC, of around 15% is due, in part, to the high rate of distant metastasis either at presentation or after therapy [14,15]. Distant metastasis can occur even after treatment has achieved local control [16,17]. Metastasis occurring after local control could be due to the growth of micrometastases already present before the start of curative-intent treatment. Indeed, disseminated tumor cells have been detected even in patients with premalignant breast ductal carcinoma *in situ* prior to the administration of any treatment [18,19]. Metastasis may also arise from uncontrolled locoregional disease. Tumor manipulation, or incomplete tumor resection before RT can also play a role [20,21]. A further possibility is that treatment itself may in some cases be responsible for the initiation of distant metastasis by inducing tumor cell dissemination through the disruption of primary tumor architecture and/or selecting for, or activating a more aggressive circulating tumor cell (CTC) phenotype [22–24].

### Metastasis & circulating tumor cells

Metastasis is a multistep process whereby cells within a primary tumor must escape from the tumor bulk, either by loss of cell–cell adhesion, physical disruption of the tumor and/or acquisition of increasing motility and invasive properties [25]. Shed tumor cells must then be able to enter the circulation, becoming CTCs, and survive until being able to extravasate into distant tissues before they have the opportunity to colonize a new site. Metastasis is an extremely inefficient process with each CTC having an extremely small probability of seeding a distant metastatic lesion [26]. However, this selection pressure increases the chance that disseminated tumor cells exhibit more aggressive phenotypes, having already evaded or resisted a number of defense mechanisms [27,28].

The collection of CTCs from the systemic circulation as a liquid biopsy is an alternative to an invasive biopsy of a single tumor location, and is considered a promising tool to examine tumor phenotype, act as a real-time biomarker, test for the presence of therapeutic targets, study treatment resistance and to increase our understanding of disease progression, metastasis, intratumor heterogeneity and cancer stem cells [29,30].

#### CTCs & prognostic significance

Several studies have evaluated the strength of CTCs as prognostic markers both in metastatic and nonmetastatic disease. In the earliest studies, a simple cut-off of greater than five CTCs isolated from 7.5 ml of peripheral blood correlated with worse progression-free survival and overall survival in studies of metastatic breast [31] and prostate cancer [32]. Evaluating CTC numbers over time in metastatic breast cancer patients showed that increased baseline CTC numbers correlated with a higher number of positive lymph nodes and increased probability of bone metastasis at baseline, and ultimately also with poor progression-free survival [33]. In fact, patients with greater than five CTCs per 7.5 ml blood at the final blood draw had a hazard ratio of 5.3 for progression-free survival compared with those with lower CTC count when included in a multivariate analysis including number of metastatic sites and positive lymph nodes. In subsequent studies, decreases in overall survival for NSCLC patients were also seen in those with greater than five CTCs per 7.5 ml blood [34]. A decrease in CTC count after treatment in patients with advanced NSCLC correlated with treatment response by conventional imaging, and greater progression-free survival [35]. The number of NSCLC patients which were CTC-positive 1 month after surgery was around half of that before surgery, while the presence of CTCs postsurgery identified NSCLC patients at risk of early disease recurrence and shorter disease-free survival [36].

#### CTCs & tumor heterogeneity

A major difficulty in the treatment of NSCLC (and indeed most cancer types) is tumor heterogeneity [37], where distinct phenotypic differences can be observed between different cancer cells within same tumor or between different tumor sites. The cause of this variability is thought to be an interaction between two processes: cancer stem cell maintenance and clonal evolution; the exact mechanisms involved in these processes have been reviewed extensively [38–41]. Tumor heterogeneity ultimately results in treatment resistance [42] due to the initial death of treatment-sensitive clones followed by repopulation from treatment-resistant clones. Methods such as genetic barcoding and red-green-blue marking have been used to track cell clonality after drug or radiation treatment of virally transduced cells to improve understanding of tumor heterogeneity and its relationship with treatment resistance [43–46]. Conceivably, such technologies could be useful for studying treatment resistance in patient-derived CTCs, where capturing the tumor cells shed into the circulation may give a better representation of tumor heterogeneity from all tumor sites than a single biopsy sample.

### Change of CTC properties: epithelial–mesenchymal transition

Epithelial–mesenchymal transition (EMT) is a normal process in particular cells types during embryonic development, but which can occur promiscuously to enhance the migratory capacity and invasiveness of tumor cells [47]. EMT-like changes, including EMT transcription factor expression and E-cadherin loss, have been observed following irradiation in a hepatocellular carcinoma (HCC) cell line and a HCC mouse model [48]. Colorectal carcinoma cell lines exposed to 5 Gy irradiation also show EMT-like changes, such as loss of cell polarity, induction of a spindle-like morphology as well as increased motility and invasiveness [49], with some evidence of corresponding EMT-like gene expression changes in pre- and post-RT tumor biopsy samples in colorectal cancer patients. Similar results were observed in a rat glioma xenograft model [50]. The surviving cells from NSCLC cell lines irradiated with 5 Gy demonstrated increased ability to be cultured as 3D spheroids [51], often used as a marker for stemness and considered an indicator of tumor-forming potential [52], with EMT known to overlap with features that define CSCs [53–55]. Other work with irradiated NSCLC cells in both cell and animal models has added further evidence of radiation-induced EMT-like changes [56], in this case mediated by autocrine release of G-CSF and induction of the JAK/STAT signaling pathway. EMT can also be induced by TGF-β signaling and mediated by matrix metalloproteinases responsible for the degradation of extracellular matrix proteins [57].

Although RT is indispensable in the attempt to control or eradicate primary NSCLC tumors, it might have the collateral effect of enhancing the metastatic potential of tumor cells shed from the primary lesion. Understanding the effect of RT on tumor cells and CTCs, and on metastasis in general, can assist in developing more effective personalized treatment and reducing the incidence of metastatic spread [58]. It should be also mentioned that other tumor interventions, such as tumor biopsy [59,60], surgical trauma [61,62] and chemotherapy [63–65] can also promote EMT and tumor cell migratory capacities.

### Isolation of CTCs from patients

#### Identifying CTCs

CTC immunophenotypes can include a mixture of exclusion markers, candidate markers, tumor hallmarks or patient-specific markers (listed from least to most specific). The simplest criteria are the exclusion markers, most notably the pan-hematopoietic cell-surface marker CD45, but also including CD2, CD16, CD19, CD36, CD38, CD66b, CD163 and CD235a [66,67]. The presence of any of these markers might be considered to denote the cell as blood- or bone marrow-derived, and thus not considered further as a potential CTC [68]. Candidate markers, most notably EpCAM and the intermediate filament proteins of the keratin family, are highly suggestive of epithelial origin and thus their presence is often considered a minimal criterion for consideration as a CTC. Tumor hallmarks can include further markers that are associated with the tumor phenotype of interest and uncommon in healthy donor blood cells and may include EGFR or HER2 expression [69], or telomerase activity [70]. These markers may help to find candidate CTCs, but might not form part of the definitive criteria. Patient-specific markers can include specific chromosomal rearrangements (detected by *in situ* hybridization) [71], mutant protein forms or biopsy-confirmed upregulated markers (such as PDL-1). Such markers are often impractical for initial screening, but might be used to validate suspected CTCs.

As the result of studies conducted by Racila *et al*. [72], Kagan *et al*. [73] and the consensus of various working groups, such as reported by van de Stolpe *et al*. [74], CTCs are typically defined as EpCAM and/or cytokeratin-positive, CD45-negative, DAPI-positive cells. However, heterogeneity between expression levels of many genes within CTCs, even within samples derived from a single patient, has been shown to be quite large suggesting that there may not be a ‘one size fits all’ approach to identifying and characterizing CTCs [75], and may cause underestimates of CTC numbers. There is recognition that such a definition may ignore relevant cells that are not yet sufficiently characterized. Where keratin expression is suspected to be reduced or lost, several tumor hallmarks or patient-specific tumor markers might be required instead to justify their inclusion. Despite their promise, CTCs are rare within the blood and thus several hurdles must be overcome for CTC isolation and manipulation.

#### Isolation based on biological properties

Positive selection for CTCs is usually performed using antibodies to CTC cell-surface markers [76]. Currently, the only method of enriching and detecting CTCs which is US FDA-approved for diagnostic use is the CellSearch CTC test system [76]. In this system, potential CTCs are enriched based on EpCAM expression, then imaged for expression of CD45 and cytokeratin. Positive selection approaches such as these risk incomplete capture of EpCAM-positive CTCs or loss of EpCAM-negative CTCs. We have shown that when CTCs were isolated from palliatively treated NSCLC patients via Ficoll density gradient centrifugation and enumerated by a manual operator counting CK+/CD45-/DAPI+ events, multiple CTCs were enumerated [22] while the CellSearch system only detected between 0–4 CTCs. However, positive selection methods can reduce the risk of detecting false-positive events by adding additional selection criteria.

Negative selection methods work by depleting the surrounding blood cells, and do not rely on CTC marker expression. The ubiquitous expression of CD45 across all hematopoietic cells makes it a useful candidate for antibody-mediated depletion and as such is the most utilized marker for this purpose [77]. Technologies building on this approach include the RosetteSep CTC enrichment cocktails [78]. These are a mixture of tetrameric antibody complexes that recognize cell-surface antigens on white blood cells and glycophorin A on erythrocytes and crosslink these cells together. These immunorosettes are depleted by density gradient centrifugation, a method shown to recover more than half of the tumor cells spiked into normal blood [79,80]. Immunomagnetic techniques employ similar antibody cocktails [81], except here unwanted cells are labeled with antibodies cross-linked to magnetic nanobeads and depleted using a magnetic field, with similar recovery of spiked tumor cells [82,83].

### Isolation based on physical properties

The physical differences of tumor cells from surrounding peripheral blood cells (deformability, size and conductivity or dielectricity) are exploited in several physical separation platforms [77].

Density gradient centrifugation is an inexpensive method of CTC enrichment. Blood is layered on a commercial modified polysaccharide solution and mononucleated cells become separated from red blood cells and granulocytes after centrifugation. Instead of using existing density gradient medium used for routine blood separation, customized media have been developed such as Oncoquick [84], to exploit the physical characteristics of tumor cells. These approaches all represent only limited enrichment of CTCs, and can provide high yields but only low purity [85,86], making the choice of downstream application important for the selection of an optimal method.

Microfiltration methods enrich CTCs on the principle that tumor cells are generally larger than the normal cells of the surrounding peripheral blood [87,88]. The original isolation of epithelial tumor cells technique (ISET) used a polycarbonate filter containing 8 μm pores to enrich CTCs from whole blood of HCC patients [89]. Since then, porous filters have been used to isolate CTCs in patients with various other cancers including NSCLC [90]. In one study, the use of ISET for CTC enrichment in metastatic breast, prostate and lung cancer outperformed the CellSearch platform [91], as have other similar filtration techniques [92,93]. The speed and high throughput of microfiltration techniques allows for rapid CTC enrichment. However, the degree of yield and enrichment is variable, and pressure/vacuum forces may affect cell viability and downstream processing [94,95].

Microfluidic approaches allow tumor cells which differ in size and deformability from normal peripheral blood cells to be enriched in channels which exploit fluid dynamics. Small pillars of differing size and shape, or crescent shaped traps within microfluidic chambers have been used to effectively isolate tumor cells from whole blood [96], as has a microfluidic approach using inertial focusing of larger tumor cells within a curvilinear channel [97]. Microfluidic approaches offer very high purity and very little disturbance of CTCs; however, these approaches can be limited in their throughput and require long processing times. A dielectrophoretic field-flow fractionation technique which separates cells based on size and/or electrical properties [98] shows promise, but currently the cells must still first undergo gradient centrifugation.

### Propagation of CTCs

The propagation of CTCs would allow expansion of cell numbers for further analysis and research. The *in vivo* propagation of CTCs using immunocompromised mice as vehicles is an approach to generate preclinical models for the study of patient tumors [99]. CTCs isolated from breast cancer patients injected directly into mouse femurs, later formed metastases in the liver, lung and bone of the mice [100]. In a later study, CTCs isolated from the blood of small-cell lung cancer patients were injected into immunocompromised mice which formed tumors at the site of injection [101]. Analysis of these xenografts showed the response to etoposide, and platinum therapy closely reflected patient overall survival.

The *ex vivo* culture of CTCs allows higher throughput and more thorough functional analysis than can be achieved in animal models. An early study described a method for isolating and culturing CTCs derived from xenografts generated from immortalized mammary tumor cells intravenously injected into mice and then recovered from the blood [102]. These CTCs were expanded short-term in standard cell culture medium containing fetal calf serum, demonstrating both the viability and *in vitro* proliferative capacity of rare CTCs isolated from the blood. Similar results were found using the same culture conditions in a later study [103], where cells from a lung cancer cell line (H460) were orthotopically implanted into mice and tumor-derived CTCs were re-isolated and cultured. These cultures exhibited a 12-h doubling time along with abnormal mitotic divisions (single cells dividing into three daughter cells). Using these same culture conditions, short-term cultures were established from patient-derived CTCs of prostate, esophageal, mesothelioma and urinary bladder cancer patients [104–107].

The establishment of long-term stable cultures has only been achieved in a handful of studies. CTCs isolated from metastatic luminal breast cancer patients senesced in culture after only several cell divisions when cultured under attached conditions in serum-containing medium [108]. Long-term CTC cultures were established from six of 35 patients when CTCs were cultured under hypoxic conditions as spheroids in serum-free media containing EGF and FGF supplements. More complex serum-free media have since been used to culture CTCs *ex vivo* [109,110]. A prostate cancer CTC line grown as organoids for over 9 months *in vitro* later formed tumors when implanted in mice [109]. In this cell line, 67% of the mutations detected were consistent with mutations that were identified within patient lymph node biopsies. The first permanent colon cancer CTC line was established by Cayrefourcq *et al*. [111] from two metastatic colorectal adenocarcinoma patients with CTC count of >300/10 ml blood. A 3D co-culture technique whereby CTCs isolated from patients using a microfluidic chip were cultured *in situ* on the capture device alongside green fluoroscent protein (GFP)-labeled cancer-associated fibroblasts, with collagen I and reconstituted basement membrane (Matrigel, NY, USA) [112]. Genetic sequencing was performed on the established CTC cultures allowing mutational landscape comparison with the primary tumor from which they arose. It has so far proven difficult to establish cell lines from CTCs of NSCLC patients but more success has been obtained with small-cell lung cancer [101].

### Possible significance of CTCs in NSCLC

While it is clear that the presence and number of CTCs and CTC clusters before and after treatment for lung cancer can have prognostic significance [113], it has not been conclusively established that mobilization of CTCs at the time of treatment independently affects outcomes in humans with cancer. Nevertheless, there has long been concern in the surgical community that CTC release during and after cancer surgery could be associated with increased rates of metastatic disease and ways to counteract this risk is actively being explored [114]. A wide spectrum of interventions ranging from tumor manipulation and biopsy to radical resection has been shown to be capable of increasing CTC count. Other events than CTC release at the time of surgery may also influence the risk of future metastasis, including anesthetic technique (inhalational vs intravenous) and the administration of various medications in the perioperative period [115]. Preclinical models of the effects of surgery on metastasis were described by Demicheli and colleagues [116], who concluded that resection was capable of affecting metastasis either positively or negatively depending on the circumstances. Folkman reported that antiangiogenic proteins such as angiostatin and endostatin, released from the primary tumor, could prevent the progression of distant metastatic lesions [117]. Resection of the primary tumor removed a brake on angiogenesis, thereby accelerating metastasis. Core needle biopsy was shown to induce distant metastasis in a preclinical system, probably due to cytokine release [59].

The effects of surgery on CTCs in patients with cancer have been most extensively studied in colorectal cancer [118]. However, there is a limited but growing surgical literature on lung cancer CTCs. Studies have shown increased levels of CTCs at the time of surgery and have demonstrated that surgical technique may influence this phenomenon. Both open and video-assisted surgical techniques are associated with increased CTC count in NSCLC [119]. It was reported by Hashimoto and colleagues that, on average, a significant increase in CTCs could be detected in the pulmonary veins in 30 patients undergoing lobectomy for NSCLC [120]. Dong and colleagues collected pulmonary venous blood in 31 NSCLC patients during open thoracic surgery. CTCs were found in 48% of patients and median survival and 2-year survival rates for patients with and without CTCs were 11 versus 27 months, and 26.7 versus 62.5%, respectively [121]. Li and colleagues [122] have suggested that ‘no touch’ techniques, such as those pioneered in colorectal and pancreatic surgery, may be able to play a role in lung cancer surgery and proposed that the approach of ligating the pulmonary veins first in video-assisted thoracoscopic lobectomy (VATS) may in part explain the superior outcomes observed with VATS compared with open resections. Data supporting this contention were reported by Huang and colleagues who studied 43 lung cancer patients treated with VATS resections and 36 who underwent conventional lobectomy, concluding that a *“smaller increase in CTCs was seen in patients treated with VATS lobectomy than in patients treated with conventional thoracotomy”* [123]. Further research is required to elucidate relationships, if any exist, between these phenomena and long term outcomes.

There is an extensive body of work in animal tumor models where investigations of correlations between local irradiation of cancers and later development of metastasis have been studied. Depending on the model and the dose and fractionation of RT, the risk of distant metastasis may increase or decrease with local RT compared with resection alone or observation (discussed in [23,24]). RT can increase the number of CTCs in some tumor models and can lead to the development of EMT in local tumors and CTCs and to metastases with EMT characteristics. Recently, using a real-time flow cytometry platform, Koonce *et al*. [124] showed that radiation, as well as nanodrug treatment triggered short-term release of CTC from the primary tumor in a mouse model. However, this release was not associated with metastasis; moreover, RT alone lead to reduction of metastasis.

The investigation of RT-associated changes in CTC numbers has only just commenced in humans with cancer in general and lung cancer in particular. Our group, as discussed above, found that in patients with more advanced NSCLC, treated with large palliative RT fractions, CTC numbers increase, including CTC clusters, in the days after commencement of treatment [22]. Many of these CTCs had evidence of high levels of DNA damage as detected by the γH2AX assay, consistent with their presence within the radiation field before shedding into the circulation. About half of patients treated with curative intent were also observed to have an increase in CTC count. The potential of systemic therapy to mobilize CTCs has not yet been systematically investigated but there is emerging evidence from animal models that CTCs can be mobilized from murine tumors in the days after immediately commencing chemotherapy [125]. In an historic study of the use of chemotherapy and G-CSF to mobilize hematopoietic stem cells prior to autologous stem cell transplantation for breast or lung cancer, increases in CTCs were observed in a high proportion of cases in the days after commencement of treatment [126].

## Conclusion

The significance of the mobilization of CTCs by cancer therapy in patients with solid tumors is currently unknown. Clinical trials currently in progress, in which CTC numbers and characteristics are serially monitored and subsequently correlated with imaging and clinical findings, may provide the answer to this question. If CTC mobilization is a potential cause of metastatic failure in a proportion of patients, then the results of therapy might be improved by targeting this phenomenon with novel therapeutic approaches.

## References

[1] Fitzmaurice C, Dicker D, Pain A (2015). The global burden of cancer 2013. *JAMA Oncol.*.

[2] Ferlay J, Soerjomataram I, Dikshit R (2015). Cancer incidence and mortality worldwide: sources, methods and major patterns in GLOBOCAN 2012. *Int. J. Cancer*.

[3] Siegel RL, Miller KD, Jemal A (2018). Cancer statistics, 2018. *CA Cancer J. Clin.*.

[4] Tanoue LT, Detterbeck FC (2009). New TNM classification for non-small-cell lung cancer. *Expert Rev. Anticancer Ther.*.

[5] Perez CA, Stanley K, Rubin P (1980). A prospective randomized study of various irradiation doses and fractionation schedules in the treatment of inoperable non-oat-cell carcinoma of the lung. Preliminary report by the Radiation Therapy Oncology Group. *Cancer*.

[6] Bradley JD, Paulus R, Komaki R (2015). Standard-dose versus high-dose conformal radiotherapy with concurrent and consolidation carboplatin plus paclitaxel with or without cetuximab for patients with stage IIIA or IIIB non-small-cell lung cancer (RTOG 0617): a randomised, two-by-two factorial Phase 3 study. *Lancet Oncol.*.

[7] Rodrigues G, Choy H, Bradley J (2015). Definitive radiation therapy in locally advanced non-small-cell lung cancer: executive summary of an American Society for Radiation Oncology (ASTRO) evidence-based clinical practice guideline. *Pract. Radiat. Oncol.*.

[8] Sause WT, Scott C, Taylor S (1995). Radiation Therapy Oncology Group (RTOG) 88–08 and Eastern Cooperative Oncology Group (ECOG) 4588: preliminary results of a Phase III trial in regionally advanced, unresectable non-small-cell lung cancer. *J. Natl Cancer Inst.*.

[9] Antonia SJ, Villegas A, Daniel D (2017). Durvalumab after chemoradiotherapy in stage III non-small-cell lung cancer. *N. Engl. J. Med.*.

[10] Goldsmith C, Gaya A (2012). Stereotactic ablative body radiotherapy (SABR) for primary and secondary lung tumours. *Cancer Imaging*.

[11] Brown WT, Wu X, Fowler JF (2008). Lung metastases treated by cyberknife image-guided robotic stereotactic radiosurgery at 41 months. *South. Med. J.*.

[12] Norihisa Y, Nagata Y, Takayama K (2008). Stereotactic body radiotherapy for oligometastatic lung tumors. *Int. J. Radiat. Oncol. Biol. Phys.*.

[13] Rusthoven KE, Kavanagh BD, Burri SH (2009). Multi-institutional Phase I/II trial of stereotactic body radiation therapy for lung metastases. *J. Clin. Oncol.*.

[14] MacManus MP, Hicks RJ, Matthews JP, Wirth A, Rischin D, Ball DL (2005). Metabolic (FDG-PET) response after radical radiotherapy/chemoradiotherapy for non-small-cell lung cancer correlates with patterns of failure. *Lung Cancer*.

[15] Germain F, Wai ES, Berthelet E, Truong PT, Lesperance M (2008). Brain metastasis is an early manifestation of distant failure in stage III nonsmall cell lung cancer patients treated with radical chemoradiation therapy. *Am. J. Clin. Oncol.*.

[16] Ahn SJ, Kim YC, Kim KS (2005). Results of curative radiation therapy with or without chemotherapy for stage III unresectable non-small cell lung cancer. *Cancer Res. Treat.*.

[17] Schytte T, Nielsen TB, Brink C, Hansen O (2014). Pattern of loco-regional failure after definitive radiotherapy for non-small cell lung cancer. *Acta Oncol.*.

[18] Hüsemann Y, Geigl JB, Schubert F (2008). Systemic spread is an early step in breast cancer. *Cancer Cell*.

[19] Sänger N, Effenberger KE, Riethdorf S (2011). Disseminated tumor cells in the bone marrow of patients with ductal carcinoma *in situ*. *Int. J. Cancer*.

[20] Juratli MA, Nedosekin DA, Sarimollaoglu M, Siegel ER, Galanzha EI, Zharov VP, Retsky MW, Demicheli R (2017). Circulating tumor cells as predictive marker in metastatic disease. *Perioperative Inflammation as Triggering Origin of Metastasis Development*.

[21] Juratli MA, Siegel ER, Nedosekin DA (2015). *In vivo* long-term monitoring of circulating tumor cells fluctuation during medical interventions. *PLoS ONE*.

[22] Martin OA, Anderson RL, Russell PA (2014). Mobilization of viable tumor cells into the circulation during radiation therapy. *Int. J. Radiat. Oncol. Biol. Phys.*.

[23] Martin OA, Anderson RL, Narayan K, MacManus MP (2016). Does the mobilization of circulating tumour cells during cancer therapy cause metastasis?. *Nat. Rev. Clin. Oncol.*.

[24] Blyth BJ, Cole AJ, MacManus MP, Martin OA (2017). Radiation therapy-induced metastasis: radiobiology and clinical implications. *Clin. Exp. Metastasis*.

[25] Hanahan D, Weinberg RA (2011). Hallmarks of cancer: the next generation. *Cell*.

[26] Fidler IJ (1970). Metastasis: quantitative analysis of distribution and fate of tumor emboli labelled with 125 I-5-iodo-2′-deoxyuridine. *J. Natl Cancer Inst.*.

[27] Jamal-Hanjani M, Wilson GA, McGranahan N (2017). Tracking the evolution of non-small-cell lung cancer. *N. Engl. J. Med.*.

[28] Swanton C, Govindan R (2016). Clinical implications of genomic discoveries in lung cancer. *N. Engl. J. Med.*.

[29] Siravegna G, Marsoni S, Siena S, Bardelli A (2017). Integrating liquid biopsies into the management of cancer. *Nat. Rev. Clin. Oncol.*.

[30] Alix-Panabières C, Pantel K (2013). Circulating tumor cells: liquid biopsy of cancer. *Clin. Chem.*.

[31] Cristofanilli M, Budd GT, Ellis MJ (2004). Circulating tumor cells, disease progression, and survival in metastatic breast cancer. *N. Engl. J. Med.*.

[32] Moreno JG, Miller MC, Gross S, Allard WJ, Gomella LG, Terstappen LW (2005). Circulating tumor cells predict survival in patients with metastatic prostate cancer. *Urology*.

[33] Nolé F, Munzone E, Zorzino L (2008). Variation of circulating tumor cell levels during treatment of metastatic breast cancer: prognostic and therapeutic implications. *Ann. Oncol.*.

[34] Krebs MG, Sloane R, Priest L (2011). Evaluation and prognostic significance of circulating tumor cells in patients with non-small-cell lung cancer. *J. Clin. Oncol.*.

[35] Punnoose EA, Atwal S, Liu W (2012). Evaluation of circulating tumor cells and circulating tumor DNA in non-small-cell lung cancer: association with clinical endpoints in a Phase II clinical trial of pertuzumab and erlotinib. *Clin. Cancer Res.*.

[36] Bayarri-Lara C, Ortega FG, Cueto Ladrón De Guevara A (2016). Circulating tumor cells identify early recurrence in patients with non-small-cell lung cancer undergoing radical resection. *PLoS ONE*.

[37] Sun XX, Yu Q (2015). Intra-tumor heterogeneity of cancer cells and its implications for cancer treatment. *Acta Pharmacol. Sin.*.

[38] Shackleton M, Quintana E, Fearon ER, Morrison SJ (2009). Heterogeneity in cancer: cancer stem cells versus clonal evolution. *Cell*.

[39] Tysnes BB (2010). Tumor-initiating and -propagating cells: cells that we would to identify and control. *Neoplasia*.

[40] Meacham CE, Morrison SJ (2013). Tumor heterogeneity and cancer cell plasticity. *Nature*.

[41] Marusyk A, Polyak K (2010). Tumor heterogeneity: causes and consequences. *Biochim. Biophys. Acta*.

[42] Burrell RA, Swanton C (2014). Tumour heterogeneity and the evolution of polyclonal drug resistance. *Mol. Oncol.*.

[43] Weber K, Thomaschewski M, Benten D, Fehse B (2012). RGB marking with lentiviral vectors for multicolor clonal cell tracking. *Nat. Protoc.*.

[44] Mohme M, Maire CL, Riecken K (2017). Optical barcoding for single-clone tracking to study tumor heterogeneity. *Mol. Ther.*.

[45] Maetzig T, Ruschmann J, Sanchez Milde L, Lai CK, Von Krosigk N, Humphries RK (2017). Lentiviral fluorescent genetic barcoding for multiplex fate tracking of leukemic cells. *Mol. Ther. Methods Clin. Dev.*.

[46] Coffey SE, Giedt RJ, Weissleder R (2013). Automated analysis of clonal cancer cells by intravital imaging. *Intravital*.

[47] Kalluri R, Weinberg RA (2009). The basics of epithelial-mesenchymal transition. *J. Clin. Invest.*.

[48] Li T, Zeng ZC, Wang L (2011). Radiation enhances long-term metastasis potential of residual hepatocellular carcinoma in nude mice through TMPRSS4-induced epithelial–mesenchymal transition. *Cancer Gene Ther.*.

[49] Kawamoto A, Yokoe T, Tanaka K (2012). Radiation induces epithelial–mesenchymal transition in colorectal cancer cells. *Oncol. Rep.*.

[50] Park JK, Jang SJ, Kang SW (2012). Establishment of animal model for the analysis of cancer cell metastasis during radiotherapy. *Radiat. Oncol.*.

[51] Gomez-Casal R, Bhattacharya C, Ganesh N (2013). Non-small-cell lung cancer cells survived ionizing radiation treatment display cancer stem cell and epithelial–mesenchymal transition phenotypes. *Mol. Cancer*.

[52] Dontu G, Abdallah WM, Foley JM (2003). *In vitro* propagation and transcriptional profiling of human mammary stem/progenitor cells. *Genes Dev.*.

[53] May CD, Sphyris N, Evans KW, Werden SJ, Guo W, Mani SA (2011). Epithelial–mesenchymal transition and cancer stem cells: a dangerously dynamic duo in breast cancer progression. *Breast Cancer Res.*.

[54] Scheel C, Weinberg RA (2012). Cancer stem cells and epithelial–mesenchymal transition: concepts and molecular links. *Semin. Cancer Biol.*.

[55] Wang SS, Jiang J, Liang XH, Tang YL (2015). Links between cancer stem cells and epithelial–mesenchymal transition. *Onco Targets Ther.*.

[56] Cui YH, Suh Y, Lee HJ (2015). Radiation promotes invasiveness of non-small-cell lung cancer cells through granulocyte-colony-stimulating factor. *Oncogene*.

[57] Lee YH, Albig AR, Regner M, Schiemann BJ, Schiemann WP (2008). Fibulin-5 initiates epithelial–mesenchymal transition (EMT) and enhances EMT induced by TGF-β in mammary epithelial cells via a MMP-dependent mechanism. *Carcinogenesis*.

[58] Francart M-E, Lambert J, Vanwynsberghe AM (2018). Epithelial–mesenchymal plasticity and circulating tumor cells: travel companions to metastases. *Dev. Dyn.*.

[59] Mathenge EG, Dean CA, Clements D (2014). Core needle biopsy of breast cancer tumors increases distant metastases in a mouse model. *Neoplasia*.

[60] Alieva M, Margarido AS, Wieles T (2017). Preventing inflammation inhibits biopsy-mediated changes in tumor cell behavior. *Sci. Rep.*.

[61] Okolie O, Bago JR, Schmid RS (2016). Reactive astrocytes potentiate tumor aggressiveness in a murine glioma resection and recurrence model. *Neuro-oncology*.

[62] Weil S, Osswald M, Solecki G (2017). Tumor microtubes convey resistance to surgical lesions and chemotherapy in gliomas. *Neuro-oncology*.

[63] Yu M, Bardia A, Wittner BS (2013). Circulating breast tumor cells exhibit dynamic changes in epithelial and mesenchymal composition. *Science*.

[64] Bhatia S, Monkman J, Toh AKL, Nagaraj SH, Thompson EW (2017). Targeting epithelial–mesenchymal plasticity in cancer: clinical and preclinical advances in therapy and monitoring. *Biochem. J.*.

[65] Latifi A, Abubaker K, Castrechini N (2011). Cisplatin treatment of primary and metastatic epithelial ovarian carcinomas generates residual cells with mesenchymal stem cell-like profile. *J. Cell. Biochem.*.

[66] Mostert B, Sleijfer S, Foekens JA, Gratama JW (2009). Circulating tumor cells (CTCs): detection methods and their clinical relevance in breast cancer. *Cancer Treat. Rev.*.

[67] Lapin M, Tjensvoll K, Oltedal S (2016). MINDEC – an enhanced negative depletion strategy for circulating tumour cell enrichment. *Sci. Rep.*.

[68] Lowes LE, Bratman SV, Dittamore R (2016). Circulating tumor cells (CTC) and cell-free DNA (cfDNA) workshop 2016: scientific opportunities and logistics for cancer clinical trial incorporation. *Int. J. Mol. Sci.*.

[69] Man Y, Wang Q, Kemmner W (2011). Currently used markers for CTC isolation – advantages, limitations and impact on cancer prognosis. *J. Clin. Exp. Pathol.*.

[70] Xu T, Lu B, Tai Y-C, Goldkorn A (2010). A cancer detection platform which measures telomerase activity from live circulating tumor cells captured on microfilter. *Cancer Res.*.

[71] Shaffer DR, Leversha MA, Danila DC (2007). Circulating tumor cell analysis in patients with progressive castration-resistant prostate cancer. *Clin. Cancer Res.*.

[72] Racila E, Euhus D, Weiss AJ (1998). Detection and characterization of carcinoma cells in the blood. *Proc. Natl Acad. Sci. USA*.

[73] Kagan M, Howard D, Bendele T (2002). A sample preparation and analysis system for identification of circulating tumor cells. *J. Clin. Ligand Assay*.

[74] Van De Stolpe A, Pantel K, Sleijfer S, Terstappen LW, Den Toonder JM (2011). Circulating tumor cell isolation and diagnostics: toward routine clinical use. *Cancer Res.*.

[75] Powell AA, Talasaz AH, Zhang H (2012). Single cell profiling of circulating tumor cells: transcriptional heterogeneity and diversity from breast cancer cell lines. *PLoS ONE*.

[76] Hsieh JCH, Wu TMH, Xu K (2016). The selection strategy for circulating tumor cells (CTCs) isolation and enumeration: technical features, methods and clinical applications. *Tumor Metastasis*.

[77] Gabriel MT, Calleja LR, Chalopin A, Ory B, Heymann D (2016). Circulating tumor cells: a review of non-EpCAM-based approaches for cell enrichment and isolation. *Clin. Chem.*.

[78] Stemcell Tech (2017). RosetteSep: CTC enrichment cocktail containing. http://www.stemcell.com/products/brands/rosettesep.html.

[79] Guo W, Yang X-R, Sun Y-F (2014). Clinical significance of EpCAM mRNA-positive circulating tumor cells in hepatocellular carcinoma by an optimized negative enrichment and qRT-PCR-based platform. *Clin. Cancer Res.*.

[80] He W, Kularatne SA, Kalli KR (2008). Quantitation of circulating tumor cells in blood samples from ovarian and prostate cancer patients using tumor-specific fluorescent ligands. *Int. J. Cancer*.

[81] Stemcell Tech (2017). EasySep: direct human CTC enrichment kit. http://www.stemcell.com/easysep-direct-human-ctc-enrichment-kit.html.

[82] Kantara C, O'Connell M, Luthra G (2015). Methods for detecting circulating cancer stem cells (CCSCs) as a novel approach for diagnosis of colon cancer relapse/metastasis. *Lab. Invest.*.

[83] Liu Z, Fusi A, Klopocki E (2011). Negative enrichment by immunomagnetic nanobeads for unbiased characterization of circulating tumor cells from peripheral blood of cancer patients. *J. Transl. Med.*.

[84] Balic M, Dandachi N, Hofmann G (2005). Comparison of two methods for enumerating circulating tumor cells in carcinoma patients. *Cytometry B Clin. Cytom.*.

[85] Gertler R, Rosenberg R, Fuehrer K, Dahm M, Nekarda H, Siewert JR, Allgayer H, Heiss MM, Schildberg FW (2003). Detection of circulating tumor cells in blood using an optimized density gradient centrifugation. *Molecular Staging of Cancer*.

[86] Harouaka RA, Nisic M, Zheng SY (2013). Circulating tumor cell enrichment based on physical properties. *J. Lab. Autom.*.

[87] Zabaglo L, Ormerod MG, Parton M, Ring A, Smith IE (2003). Cell filtration-laser scanning cytometry for the characterisation of circulating breast cancer cells. *Cytometry A*.

[88] Vona G, Sabile A, Louha M (2000). Isolation by size of epithelial tumor cells: a new method for the immunomorphological and molecular characterization of circulating tumor cells. *Am. J. Pathol.*.

[89] Fehm T, Solomayer EF, Meng S (2005). Methods for isolating circulating epithelial cells and criteria for their classification as carcinoma cells. *Cytotherapy*.

[90] Vona G, Sabile A, Louha M (2000). Isolation by size of epithelial tumor cells: a new method for the immunomorphological and molecular characterization of circulating tumor cells. *Am. J. Pathol.*.

[91] Hofman V, Bonnetaud C, Ilie MI (2011). Preoperative circulating tumor cell detection using the isolation by size of epithelial tumor cell method for patients with lung cancer is a new prognostic biomarker. *Clin. Cancer Res.*.

[92] Farace F, Massard C, Vimond N (2011). A direct comparison of CellSearch and ISET for circulating tumour-cell detection in patients with metastatic carcinomas. *Br. J. Cancer*.

[93] Zheng S, Lin H, Liu J-Q (2007). Membrane microfilter device for selective capture, electrolysis and genomic analysis of human circulating tumor cells. *J. Chromatogr. A*.

[94] Desitter I, Guerrouahen BS, Benali-Furet N (2011). A new device for rapid isolation by size and characterization of rare circulating tumor cells. *Anticancer Res.*.

[95] Yu M, Stott S, Toner M, Maheswaran S, Haber DA (2011). Circulating tumor cells: approaches to isolation and characterization. *J. Cell Biol.*.

[96] Pugia M, Magbanua MJM, Park JW, Magbanua MJM, Park JW (2017). Enrichment and detection of circulating tumor cells and other rare cell populations by microfluidic filtration. *Isolation and Molecular Characterization of Circulating Tumor Cells*.

[97] Tan SJ, Yobas L, Lee GY, Ong CN, Lim CT (2009). Microdevice for the isolation and enumeration of cancer cells from blood. *Biomed. Microdevices*.

[98] Hou HW, Warkiani ME, Khoo BL (2013). Isolation and retrieval of circulating tumor cells using centrifugal forces. *Sci. Rep.*.

[99] Gupta V, Jafferji I, Garza M (2012). ApoStream(™), a new dielectrophoretic device for antibody independent isolation and recovery of viable cancer cells from blood. *Biomicrofluidics*.

[100] Pretlow TG, Schwartz S, Giaconia JM (2000). Prostate cancer and other xenografts from cells in peripheral blood of patients. *Cancer Res.*.

[101] Baccelli I, Schneeweiss A, Riethdorf S (2013). Identification of a population of blood circulating tumor cells from breast cancer patients that initiates metastasis in a xenograft assay. *Nat. Biotechnol.*.

[102] Hodgkinson CL, Morrow CJ, Li Y (2014). Tumorigenicity and genetic profiling of circulating tumor cells in small-cell lung cancer. *Nat. Med.*.

[103] Kang JH, Krause S, Tobin H, Mammoto A, Kanapathipillai M, Ingber DE (2012). A combined micromagnetic-microfluidic device for rapid capture and culture of rare circulating tumor cells. *Lab Chip*.

[104] Kolostova K, Zhang Y, Hoffman RM, Bobek V (2014). *In vitro* culture and characterization of human lung cancer circulating tumor cells isolated by size exclusion from an orthotopic nude-mouse model expressing fluorescent protein. *J. Fluoresc.*.

[105] Kolostova K, Broul M, Schraml J (2014). Circulating tumor cells in localized prostate cancer: isolation, cultivation *in vitro* and relationship to T-stage and Gleason score. *Anticancer Res.*.

[106] Bobek V, Matkowski R, Gürlich R (2014). Cultivation of circulating tumor cells in esophageal cancer. *Folia Histochem. Cytobiol.*.

[107] Bobek V, Kacprzak G, Rzechonek A, Kolostova K (2014). Detection and cultivation of circulating tumor cells in malignant pleural mesothelioma. *Anticancer Res.*.

[108] Cegan M, Kolostova K, Matkowski R (2014). *In vitro* culturing of viable circulating tumor cells of urinary bladder cancer. *Int. J. Clin. Exp. Pathol.*.

[109] Yu M, Bardia A, Aceto N (2014). *Ex vivo* culture of circulating breast tumor cells for individualized testing of drug susceptibility. *Science*.

[110] Gao D, Vela I, Sboner A (2014). Organoid cultures derived from patients with advanced prostate cancer. *Cell*.

[111] Kulasinghe A, Perry C, Warkiani ME (2016). Short term *ex vivo* expansion of circulating head and neck tumour cells. *Oncotarget*.

[112] Cayrefourcq L, Mazard T, Joosse S (2015). Establishment and characterization of a cell line from human circulating colon cancer cells. *Cancer Res.*.

[113] Zhang Z, Shiratsuchi H, Lin J (2014). Expansion of CTCs from early stage lung cancer patients using a microfluidic co-culture model. *Oncotarget*.

[114] Wang J, Wang K, Xu J, Huang J, Zhang T (2014). Prognostic significance of circulating tumor cells in non-small-cell lung cancer patients: a meta-analysis. *PLoS ONE*.

[115] Behrenbruch C, Shembrey C, Paquet-Fifield S (2018). Surgical stress response and promotion of metastasis in colorectal cancer: a complex and heterogeneous process. *Clin. Exp. Metastasis*.

[116] Dubowitz JA, Sloan EK, Riedel BJ (2017). Implicating anaesthesia and the perioperative period in cancer recurrence and metastasis. *Clin. Exp. Metastasis*.

[117] Demicheli R, Retsky MW, Hrushesky WJ, Baum M, Gukas ID (2008). The effects of surgery on tumor growth: a century of investigations. *Ann. Oncol.*.

[118] Holmgren L, O'Reilly MS, Folkman J (1995). Dormancy of micrometastases: balanced proliferation and apoptosis in the presence of angiogenesis suppression. *Nat. Med.*.

[119] Peach G, Kim C, Zacharakis E, Purkayastha S, Ziprin P (2010). Prognostic significance of circulating tumour cells following surgical resection of colorectal cancers: a systematic review. *Br. J. Cancer*.

[120] Hermansson U, Konstantinov IE, Arén C (1997). Tumor dissemination after video-assisted thoracic surgery: What does it mean?. *J. Thorac. Cardiovasc. Surg.*.

[121] Hashimoto M, Tanaka F, Yoneda K (2014). Significant increase in circulating tumour cells in pulmonary venous blood during surgical manipulation in patients with primary lung cancer. *Interact. Cardiovasc. Thorac. Surg.*.

[122] Dong Q, Huang J, Zhou Y (2002). Hematogenous dissemination of lung cancer cells during surgery: quantitative detection by flow cytometry and prognostic significance. *Lung Cancer*.

[123] Li WW, Klomp HM, Van Boven WJ, De Mol BA (2014). eComment. Circulating tumour cells caused by surgical manipulation in patients with lung cancer. Is minimally invasive “no-touch” surgery the solution?. *Interact. Cardiovasc. Thorac. Surg.*.

[124] Huang HB, Ge MJ (2016). The effects of different surgical approaches on the perioperative level of circulating tumor cells in patients with non-small-cell lung cancer. *Thorac. Cardiovasc. Surg.*.

[125] Koonce NA, Juratli MA, Cai C (2017). Real-time monitoring of circulating tumor cell (CTC) release after nanodrug or tumor radiotherapy using *in vivo* flow cytometry. *Biochem. Biophys. Res. Commun.*.

[126] Brugger W, Bross K, Frisch J (1992). Mobilization of peripheral blood progenitor cells by sequential administration of interleukin-3 and granulocyte-macrophage colony- stimulating factor following polychemotherapy with etoposide, ifosfamide, and cisplatin. *Blood*.

